# Identification of a Novel Parallel β‐Strand Conformation within Molecular Monolayer of Amyloid Peptide

**DOI:** 10.1002/advs.201500369

**Published:** 2016-04-08

**Authors:** Lei Liu, Qiang Li, Shuai Zhang, Xiaofeng Wang, Søren Vrønning Hoffmann, Jingyuan Li, Zheng Liu, Flemming Besenbacher, Mingdong Dong

**Affiliations:** ^1^ Institute for Advanced MaterialsJiangsu UniversityZhenjiang212013P. R. China; ^2^ Interdisciplinary Nanoscience Center (iNANO)Aarhus UniversityAarhus CDK‐8000Denmark; ^3^ Key Laboratory for Biomedical Effects of Nanomaterials and NanosafetyInstitute of High Energy PhysicsChinese Academy of SciencesBeijing100049P. R. China; ^4^ Department of Physics and AstronomyAarhus UniversityAarhus CDK 8000Denmark; ^5^ Center for Programmable MaterialsSchool of Materials Science and EngineeringNanyang Technological University SingaporeSingapore639798Singapore

**Keywords:** amyloid peptide aggregation, atomic force microscopy, secondary structure of amyloid peptide

## Abstract

The differentiation of protein properties and biological functions arises from the variation in the primary and secondary structure. Specifically, in abnormal assemblies of protein, such as amyloid peptide, the secondary structure is closely correlated with the stable ensemble and the cytotoxicity. In this work, the early Aβ_33‐42_ aggregates forming the molecular monolayer at hydrophobic interface are investigated. The molecular monolayer of amyloid peptide Aβ_33‐42_ consisting of novel parallel β‐strand‐like structure is further revealed by means of a quantitative nanomechanical spectroscopy technique with force controlled in pico‐Newton range, combining with molecular dynamic simulation. The identified parallel β‐strand‐like structure of molecular monolayer is distinct from the antiparallel β‐strand structure of Aβ_33‐42_ amyloid fibril. This finding enriches the molecular structures of amyloid peptide aggregation, which could be closely related to the pathogenesis of amyloid disease.

## 1 Introduction

Protein structures and properties are closely correlated to the functionality. In most cases, the differing biological functions arise from the variation in the primary and secondary structures of protein.^1^ A variety of physical properties of proteins are related to their different secondary structures. For instance, the conformation of protein in native silk fibers is an antiparallel β‐sheet, as for the silk film extracted from the glands is primarily α‐helical, which results in the totally different mechanical performance.^1^ In the case of abnormal assemblies of protein, the variation of secondary structure of these misfolded proteins or even the conformation rearrangement will trigger the changing of physicochemical properties and cause amyloid‐related diseases. For example, the cross β‐structure for the prion protein in the infectious aggregates induces the conformation conversion from α‐helical to cross β‐structure.^2^ The secondary structure conversion in protein does facilitate the amyloid‐like fibril formation that is related to the pathogenesis of amyloid diseases.^2^, ^3^ In the past few years, the oligomers of amyloid peptide have been extensively studied, and the strong evidence for the extreme polymorphism of amyloid oligomers with the cytotoxicity was provided,^4^ such as oligomers,^5^ nanopores,^6^ and other soluble amyloid β‐barrel. These amyloid oligomers are considered to cause serious damage to the cell.^7^ Moreover, the secondary structures of amyloid oligomers are distinct from amyloid fibrils. The β‐barrel oligomers of αB‐crystallin (a chaperone protein) with antiparallel β‐sheet conformation are more toxic than the amyloid fibrils of αB‐crystallin with β hairpin structure.^4^ The oligomer of Aβ_42_ composed of loosely aggregated strands with a turn conformation is also neurotoxic, which does not have the β‐sheet structure characteristic of fibrils.[[qv: 5c]] In biological system, the surface of cell membrane would be the target for amyloid oligomers. Moreover, the mechanism of amyloid peptide assembly was revealed to link to the adsorption of peptide onto molecular surfaces, such as cell membrane and other macromolecule surfaces.^8^ The surface can bind peptides, increase the local concentrations, and modulate the interchain interaction, which will result in the modulation of peptide self‐assembled nanostructure. To explore the pathway of amyloid peptide aggregation, the artificial surface was utilized to simplify and mimic the environment where the protein misfolded. The aggregation mechanism of amyloid peptides and its relation to the pathological process by surface mediation can be expounded. Recently, many surfaces have been demonstrated to be able to mediate the amyloid fibril formation, including nanoparticles,^9^ polymeric films,^10^ charged mica,^11^ and graphite.^12^ For instance, on graphite, the lamella structure of assembled peptides with antiparallel β‐sheet secondary structure was revealed by scanning tunneling microscopy (STM), which is proposed to be closely related to the amyloid fibrillization.^13^

In the case of amyloid peptide fragment originating from residue 703–712 (Aβ_33‐42_) of the amyloid precursor protein (APP), it can self‐assemble into amyloid fibrils adopting antiparallel β‐sheet secondary structure, which is figured out by STM and Fourier transform infrared (FT‐IR) spectroscopy. Solid‐state NMR has also revealed the antiparallel β‐sheet secondary in the similar case of residue 704–712 of APP. In this work, the different structures of Aβ_33‐42_ aggregation were revealed by introducing different surfaces, e.g., graphite, graphene oxide (GO). Molecular monolayer of amyloid peptide was identified by means of a new quantitative nanomechanical spectroscopy technique with force controlled in pico‐Newton (pN) range that is capable of obtaining nanoscale resolution of individual molecule or supramolecular structures.^14^ Most significant is that the molecular monolayer consists of peptide nanostripes with parallel β‐strand‐like configuration dominated by hydrophobic interactions between peptides. These nanostrips are distinct from the antiparallel β‐sheet structure in which more intermolecular hydrogen bonds involving in amyloid fibrillation. These findings were also supported by molecular dynamic (MD) simulation. The variation of secondary structure of amyloid peptides or even the conformation rearrangement explored in this work might trigger the changing of ensemble properties. The newly identified assembly of amyloid peptides by the mediation of hydrophobic surface is significant to get the insight into the pathogenesis of amyloid disease.

## 2 Results and Discussion

### 2.1 Fibrillation of Aβ_33‐42_ Peptide with Antiparallel β‐Strand Secondary Structure

**Figure 1:**
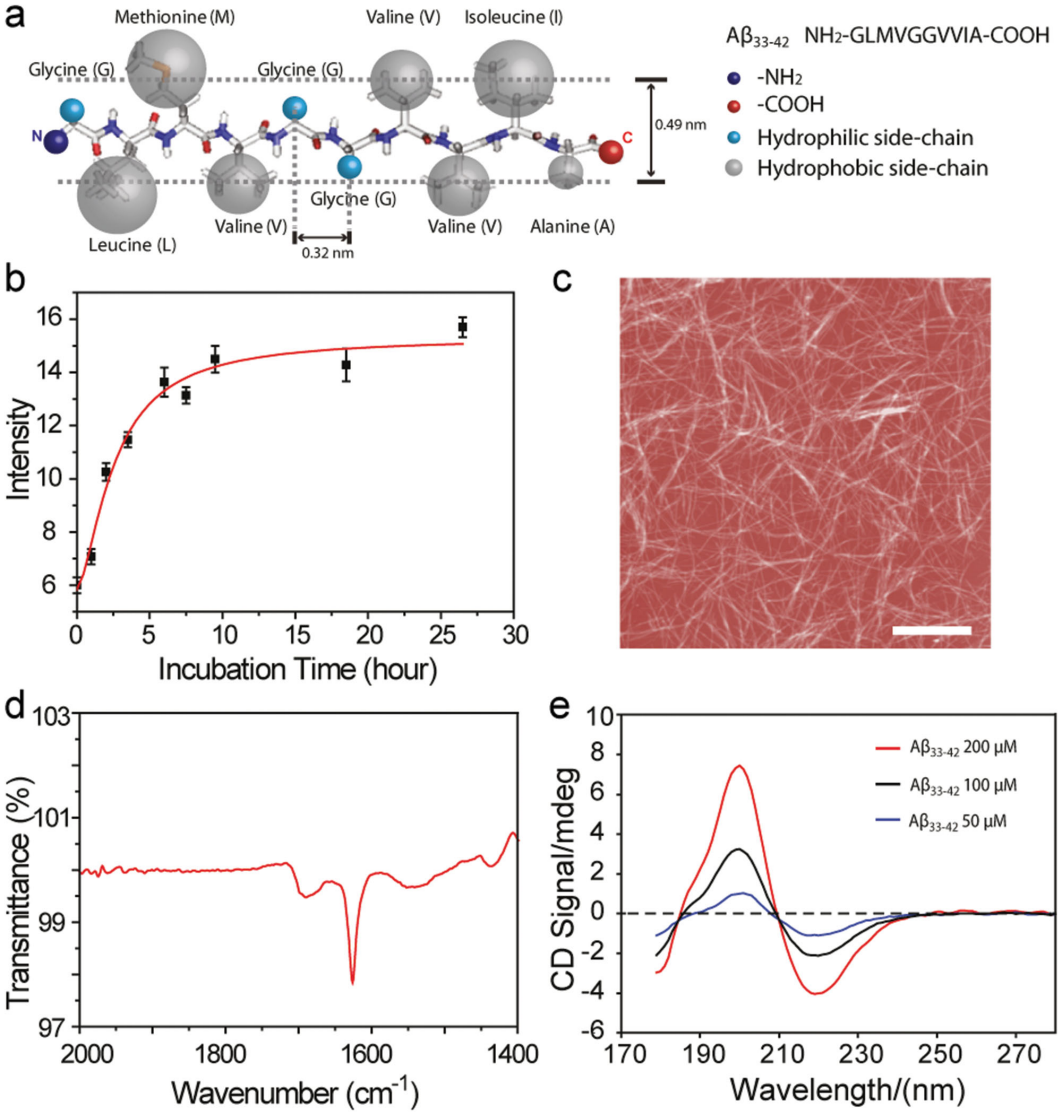
Fibrillation and the secondary structure of Aβ_33‐42_ peptide. a) Schematic model of Aβ_33‐42_; the sequence of Aβ_33‐42_ is on the top right corner and the hydrophilic and hydrophobic residues are labeled as blue and gray balls; the average width of protein and the separation of two neighboring residues are 0.49 and 0.32 nm, respectively. b) The aggregation behavior of Aβ_33‐42_ in aqueous solution as measured by Turbidity measurement. c) The amyloid fibrils assembled from Aβ_33‐42_ peptide. d) The FT‐IR spectra of Aβ_33‐42_ peptide contributing to the fibrils. e) SRCD spectra of Aβ_33‐42_. The scan range is from 280 to 170 nm. The positive peak at 198 nm and negative peak at 218 nm is the representative feature of β‐sheet secondary conformation of peptide contributing to the fibril formation. Red, black, and blue curves are the peaks of Aβ33‐42 at concentrations of 200, 100, and 50 × 10^−6^ m, respectively. Scale bar is 2 μm.

Aβ_33‐42_ originates from residue 703–712 of the APP.^15^ This short peptide is a protein segment located in the transmembrane domain of APP that is an integral membrane protein that attaches to biological membranes.^16^ This segment consists of seven hydrophobic residues and three hydrophilic residues (**Figure** **1**a), where the hydrophobic residues make up the majority of the segment. The separation between two neighboring residues is estimated to be 0.32 nm and the average height of the peptide is estimated to be 0.49 nm.^17^ The peptides with a concentration of 100 × 10^−6^ m possess the typical aggregation behavior of amyloid peptide in aqueous solution, which was revealed by the growing curve through turbidity analysis (Figure 1b). The structures of the peptide aggregates in bulk solution are typical fibril morphology (Figure 1c). The further insight into the secondary conformation of mature Aβ_33‐42_ fibrils was determined to be antiparallel β‐strand by FT‐IR spectroscopy, and the major peak at 1628 cm^−1^ with minor shoulder peak at 1696 cm^−1^ (Figure 1d) can serve as the typical characteristic.^18^ It is also nearly consistent with the previous report of anti‐parallel β‐strand for Aβ_34‐42_ amyloid fibrils.^19^ Interestingly, the short time incubation (30 min) of Aβ_33‐42_ was determined to be typical β‐strand secondary structure (Figure 1e) by using synchrotron radiation circular dichroism (SRCD) spectroscopy,^20^ which is independent of the concentration from 200 to 50 × 10^−6^ m. In the relation to the pathological process, the surface was always involved through the amyloid assembly. It is of utmost significance to introduce the artificial surface to reveal the pathway of amyloid peptide assembly.

### 2.2 Assembled Molecular Monolayer of Aβ_33‐42_ Peptide Was Formed in the Mediation of Interface

**Figure 2:**
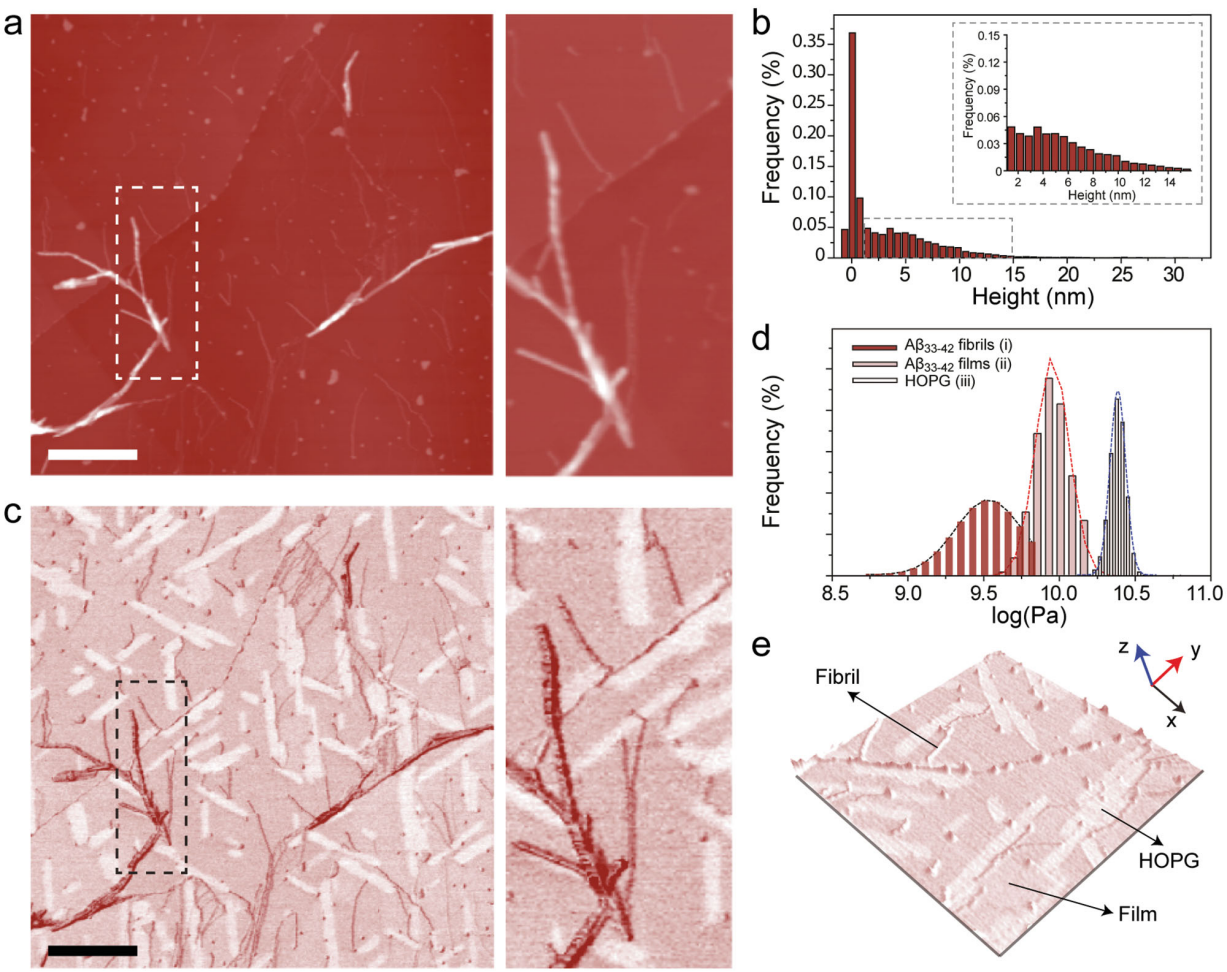
Quantitative nanomechanical images of the assembled nanostructure of Aβ_33‐42_ peptide by the mediation of carbon surface. a) The topography map of Aβ_33‐42_ assembly structure on HOPG surface. The high‐resolution map of Aβ_33‐42_ assembly is on the right panel of (a). b) The height histogram of selected Aβ_33‐42_ assemblies; the dominant distribution from the background is about 0 nm, which is a HOPG surface. The zoom‐in plot shows the height histogram of selected peptide assemblies assigned from 1 to 15 nm. c) Stiffness map of Aβ_33‐42_ assemblies and high‐resolution image on the right panel of (c). The scale bar in (a) and (c) is 1 μm. d) The different stiffness distributions correspond to Aβ_33‐42_ fibrils, Aβ_33‐42_ amyloid molecular monolayer, and HOPG substrate obtained from the stiffness map of peptide assemblies The 3D overlap map of topography and stiffness images of Aβ_33‐42_. The axis *x*, *y*, and *z* are displayed in (e) and scan size, 2 μm.

The solid surface was utilized to mediate the peptide adsorption, binding and molecular conformation rearrangement. Hence, fresh amyloid peptide solution is directly applied onto graphite surface, which allows for the peptide aggregation, whereby it mimics a naturally occurring process where amyloid peptides accumulate on the hydrophobic interface environment. The mediated self‐assembled structure of Aβ_33‐42_ was identified by quantitative nanomechanical mapping with force controlled in pN range.^14^, ^21^ The topography image (**Figure** **2**a) reveals three components, including the fibrils, a few spherical oligomers, and the substrate. Figure 2b is the corresponding height histogram for the selected area, from which it can be found that the height of 0 nm peak is the background substrate. The height of amyloid fibrils was determined to be ≈5 nm (Figure S1, Supporting Information). It is obviously noticed that there must exist other amyloid nanostructures except amyloid oligomers contributing to the height histogram below 5 nm (Figure 2b). The in situ recorded stiffness map of amyloid peptide aggregates allows us to determine Young's modulus data for the sample, and three species with different contrast (Figure 2c) can be distinguished clearly by this way. The fibrils and spherical oligomers are directly identified from their morphology, and they possess the lowest contrast in the stiffness map compared to the other species. The brightest and therefore the highest contrast is attributed to the substrate as the stiffness of graphite ranging from 18 to 56 GPa,^22^ much higher than that of peptide assemblies such as nanofibrils.^23^ The areas of medium contrast are easily identified from substrate and likely a peptide molecular monolayer structure. The stiffness of three species is also studied and their distributions are well separated (Figure 2d). Three peaks represent the relative stiffness of (i) spherical oligomers and nanofibrils (3.2 ± 1.1 GPa), (ii) a newfound peptide molecular monolayer (9.6 ± 0.6 GPa), and (iii) graphite (24.0 ± 2.8 GPa), respectively. The stiffness of the amyloid fibrils determined is consistent with the ones in the previous researches.^24^ The newfound molecular monolayer structure is three times stiffer than conventional amyloid fibrils and spherical oligomers. The increase in stiffness can be explained by both the structures and the proximity of the new species to the solid substrate, and the substrate gives some contribution to increasing stiffness, which implied that the assembled structure is a kind of molecular layer close to the hydrophobic interface. The coverage area of three different species (i = 5.2%; ii = 70.9%; iii = 23.9%) determined from Figure 2c demonstrates that the peptide molecular monolayer (ii) is the dominant nanostructure (70.9% surface coverage) in comparison with the spherical oligomers and fibrillary structures (total 5.2%, i). Figure 2e shows the overlap of surface topography and stiffness map for the peptide assembled nanostructures, which provides a correlation between the assembled structure and nanomechanical properties.

### 2.3 Molecular Monolayer of Aβ_33‐42_ Peptide Assembled from Peptide Nanostripe

**Figure 3:**
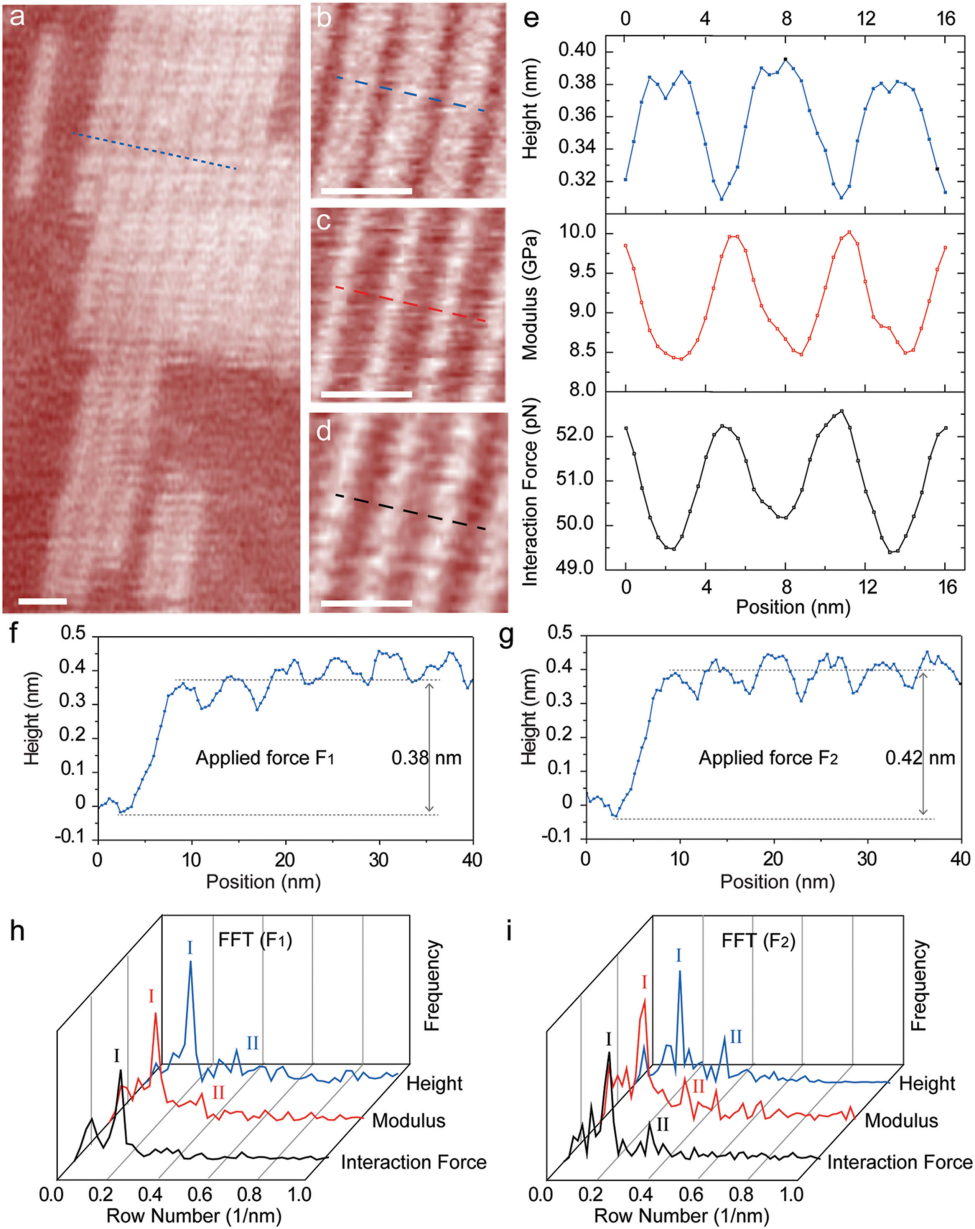
High‐resolution nanomechanical map of amyloid molecular monolayer. a) Topography map of Aβ_33‐42_ nanostriped structures, scale bar = 10 nm. b) Height, c) elastic modulus, and d) interaction force maps of amyloid peptide nanostripes. Scale bar = 10 nm in (b–d). e) Numerical values of height, modulus, and interaction force plotted from left to right across the dashed lines in b–d, respectively. All the maps were obtained under an applied force F_1_. f,g) The line profile along the dashed line marked in (a) under applied force of F_1_ and F_2_, respectively. h,i) The Fourier transformation analysis of height, modulus, and force maps of nanostripe structures under applied force of F_1_ and F_2_, respectively. Peak I and Peak II represent the two different periodicities in the nanostripe structures of amyloid peptides.

To further investigate the fine structures of amyloid molecular monolayer, we correlated the 2D surface topography with the Young's modulus and interaction force mapping under a control force in the pN range. **Figure** **3**a shows the high‐resolution topography image of peptide assemblies, where the peptide molecular monolayer is found to be composed of nanostriped structures that may be ascribed to the assemblies of amyloid peptide with β‐strand configuration. To ensure the observed features and obtain insight into the properties of this amyloid peptide assembly, the Young's modulus and interaction force maps were recorded in real time with high lateral resolution. Compared to the topography image (Figure 3b), the nanostriped structures with periodic feature were also revealed in the in situ Young's modulus (Figure 3c) and force interaction maps (Figure 3d) but presenting an inverse imaging contrast (Figure 3e). It is indicated that the dark region represents amyloid nanostripes, whereas the bright regions represent the boundary between two stripes. We calculated the stiffness of the peptide nanostripes on the 2D graphite surface to be about 9.6 GPa (Figure 3e). The vertical values of force and stiffness are based on multiple force wave force curves. Furthermore, when the imaging force was in situ decreased to half, the height of the peptide nanostripes was changed from 0.38 ± 0.01 nm (Figure 3f: F**_1_**) to 0.42 ± 0.02 nm (Figure 3g: F_2_ = F_1_/2) (Figure S2, Supporting Information). This is consistent with the expected width of the peptide displayed in Figure 1a. The height flexibility of peptide stripe is attributed to the compression of peptide residues under different applied imaging forces. We tentatively suggest that the amyloid peptide molecular monolayers we observed are molecular monolayers, which are likely to be ones mediated by hydrophobic interface.

In addition, the periodicity of the observed amyloid peptide naonstripes was explored by the fast Fourier transformation (FFT) analysis of the assembled structures in height, stiffness, and interaction maps (Figure 3h,i). When either F_1_ or F_2_ force is applied to the sample, two peaks are visible in the FFT plots (Figure S3, Supporting Information) representing the height and Young's modulus. However, the force difference can result in disappearance of peak I, indicating the deformable property of the peptide molecular monolayer. In the FFT plots, every peak represents a single periodicity. The periodicities of the nanostripe under varying applied forces are depicted (Table S1, Supporting Information). Two periodicities are estimated to be ≈2.81 ± 0.11 and 5.50 ± 0.10 nm, respectively, which are consistent with the expected lengths of one peptide and a peptide dimer, respectively. The real building blocks of the stripes are likely to be the peptide dimers and we have carried out theoretical calculations by density‐functional theory to obtain further insight into the dimer interactions. The contrast in the interaction force map determines the force difference between atomic force microscopy (AFM) tip and the peptide stripe structures, and the similar one has been done by AFM in bimodal dynamic force microscopy.^25^ The top of nanostripe structures and the boundary of nanostripe structures can be easily identified due to contact area and wettability variable between the top of nanostripe structure and the boundary of two stripes. Two possible interactions (Figure S4, Supporting Information) have been modeled and the head‐to‐head configuration with interaction between C‐termini (C—C) is more stable than the head‐to‐tail configuration with interaction between C‐terminus and N‐terminus (C—N). The dimers formed through C‐termini are energetically favorable conformation (Table S2, Supporting Information). The binding energy of the two carboxylic groups in C—C configuration is about −0.39 eV, which is considerably more stable than C—N configuration (−0.06 eV). The tail–tail interaction is mainly from van der Waals (VdW) force. Such interaction is long‐range force compared to hydrogen bonding. Therefore, the tail–tail interaction will contribute to the contrast for the boundary in the image.

### 2.4 The Theoretical Simulation of Parallel β‐Strand‐Like Conformation of Aβ_33‐42_ within Molecular Monolayer by the Interface Mediation

**Figure 4:**
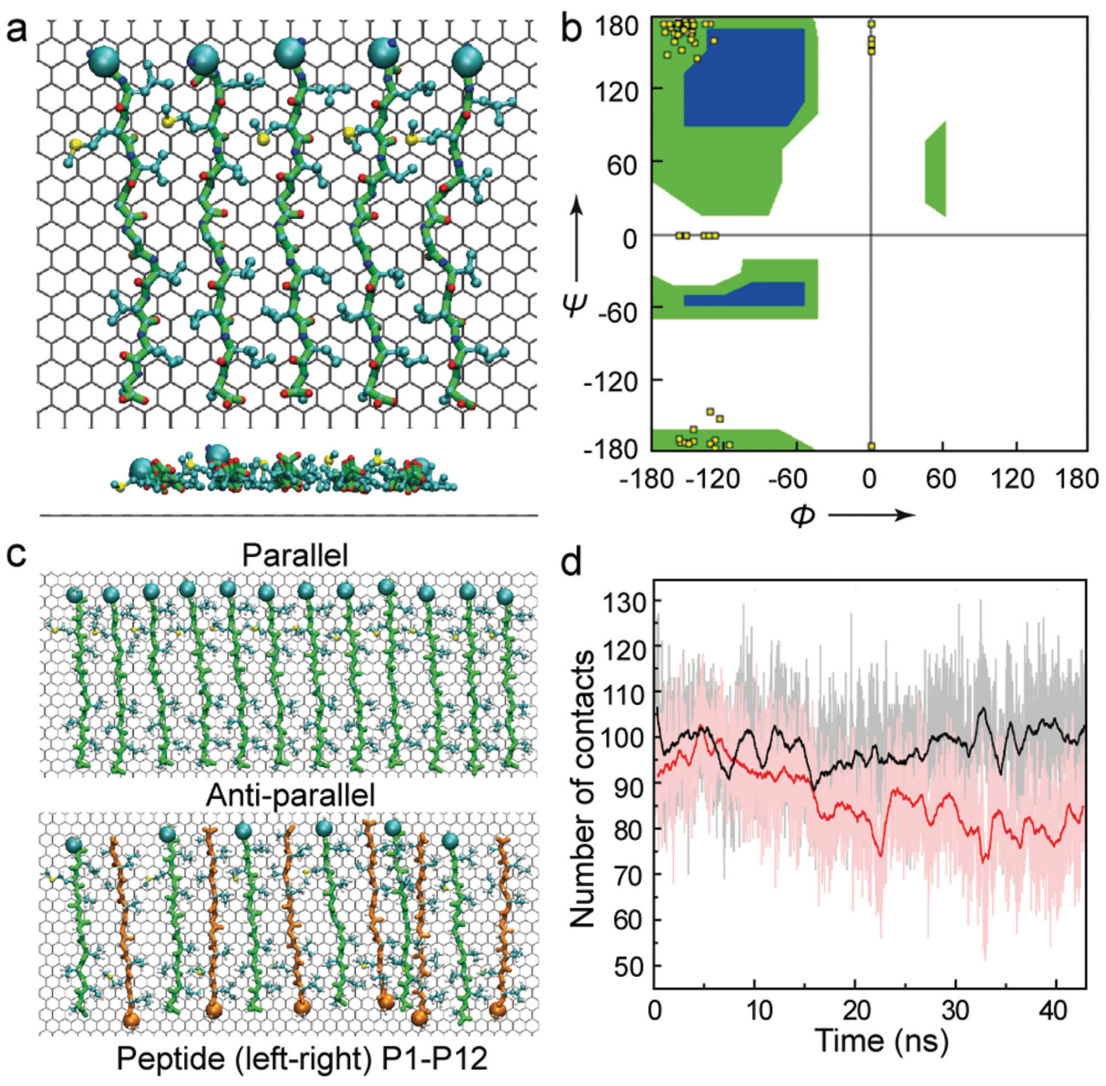
The theoretical simulation of the self‐assembly of Aβ_33‐42_ peptide on graphene. a) The configuration of five Aβ peptides adsorbed on graphene (top view, side view). C‐alpha atoms of first residues are depicted as spheres. b) The Ramachandran plot of Aβ peptides. c) The configurations of 12 Aβ peptides adsorbed on graphene, in parallel and antiparallel configurations separately. In antiparallel packing assembly, the peptides are colored green and orange alternatively. d) The number of atom contacts among four peptides in the middle part of assembly (rectangle region in (c)). The raw data are presented as background and the solid line is 100 points smoothed.

Further, MD simulation was used to study the conformation and stability of amyloid peptide assemblies by the graphene mediation. Aβ_33‐42_ peptide can quickly adsorb onto the graphene. Within the assembly, linear configurations were obtained. The side chain of all hydrophobic residues can fully adsorb to the graphene surface (**Figure** **4**a). The backbone dihedral angle was calculated and the secondary conformation of such adsorbed peptide is still β‐strand according to Ramachandran plot (Figure 4b), but the spacing between two peptides is set to 7.5 Å based on the conformation of fully adsorbed peptide. With such separation, the hydrophobic interaction between the hydrophobic residues of neighboring peptides can dominantly contribute to the stability of peptide assemblies. The side chain of hydrophobic residues of adjacent peptides can insert to each other. And the hydrogen bond between the backbones is prohibited given by such interpeptide separation. It is important to note that the mechanism of the assembly formed by adsorbed Aβ_33‐42_ is largely attributed to the hydrophobic interaction, in sharp contrast to many other peptide assemblies where the backbone hydrogen bond is prevailing and contributes to the assembly stability. More importantly, the packing orientation of amyloid peptide in the assembly is ought to determine the assembly arrangement, i.e., the parallel or antiparallel peptide assembly. We constructed the assembly formed by 12 Aβ_33‐42_ peptides (denoted to peptide P_1_ to P_12_). The interpeptide separation is also set to 7.5 Å (Figure 4c). The peptides at both ends of assembly are restrained to their initial position. The parallel orientated assembly remains very stable during 40 ns MD simulation, while the structure of antiparallel orientated assembly undergoes considerable fluctuation. The structural stability of four peptides in the middle region (i.e., peptide P_5_, P_6_, P_7_, P_8_) is further estimated by the number of contacted atom pairs formed by neighboring peptides (i.e., peptide pair P_5_‐P_6_, P_6_‐P_7_, P_7_‐P_8_). The average number of contact atom pairs in parallel orientated structure (based on last 20 ns trajectory) is 99, while such number decreases to 80 in the case of antiparallel orientated structure (Figure 4d). The parallel orientated assembly is relatively more stable than the antiparallel orientated assembly. It is consistent with the experimental observation on graphite surface and the proposed models about the structure of multiple amyloid peptide assemblies.

### 2.5 Suspended GO Mediates Aβ_33‐42_ to Form Parallel β‐Strand‐Like Molecular Monolayer

**Figure 5:**
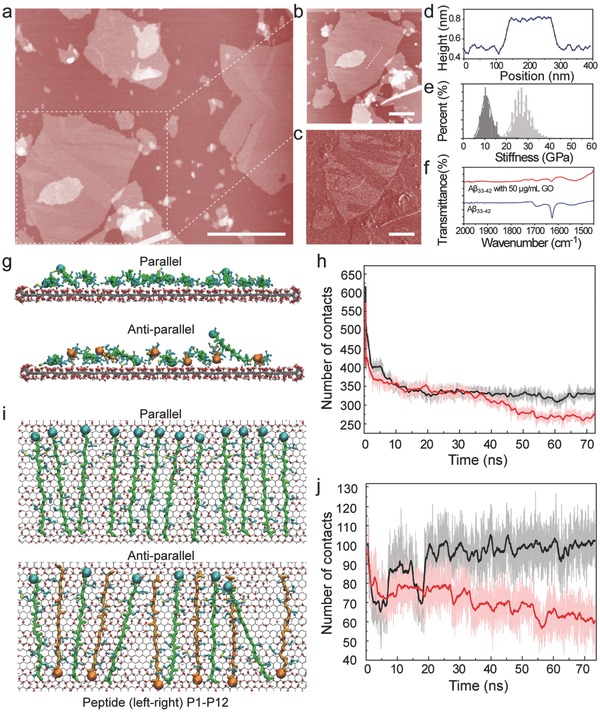
GO mediates Aβ_33‐42_ to form molecular monolayer and the corresponding theoretical simulation. a) The topography image of the molecular monolayer of Aβ_33‐42_ in the mediation of GO. b) The high‐resolution topography image of the molecular monolayer formed by Aβ_33‐42_. c) The stiffness map of the molecular monolayer of Aβ_33‐42_ on GO. d) The height profile of Aβ_33‐42_ monolayer structure on GO. e) The stiffness histograms of Aβ_33‐42_ monolayer structure on GO and of GO. The scale bar is 1 μm in (a), and 0.5 μm in (b,c). f) The secondary structure of Aβ_33‐42_ and Aβ_33‐42_ monolayer on GO by FT‐IR spectra. g–j) Detailed analysis of MD runs for 12 peptide chains, where the parallel packing peptides and antiparallel packing peptides are adsorbed on same GO. g,i) Final configuration of parallel and antiparallel packing assembly adsorbed on GO (side view, (g); top view, (i)). The color scheme is similar to that in Figure 4. h) The number of heavy atoms in direct contact with GO. j) The number of atom contacts among four peptides in the middle part of assembly. The raw data are presented as background and the solid line is 100 points smoothed.

To further verify the secondary conformation of amyloid peptide in the molecular monolayer structure in bulk solution, biocompatible GO was introduced because GO with the hydrophobic/hydrophilic domain was considered to be a good candidate as the biomimetic membrane. The basic characteristics of GO are presented in Figure S5, Supporting Information. The height of GO and the Raman spectra of GO we obtained were consistent with the previous studies.^26^ The amyloid peptide was incubated for 8 h at 37 °C with GO, and finally the complex was obtained. Some molecular monolayers of amyloid peptide were formed at the surface of GO, which was observed by AFM‐based force spectroscopy (**Figure** **5**a). The high‐resolution topography image and height profile (≈0.35 nm) are shown in Figure 5b,d, which indicates that amyloid peptides could also assemble into molecular monolayer at GO surface. The stiffness map of the complex further identified two ingredients with different elastic modulus (Figure 5c,e). The stiffness of amyloid peptide molecular monolayer was consistent with the one obtained at the surface of graphite. Therefore, it is clearly indicated that the structure formed by amyloid peptides at GO surface in bulk solution is the same as the one observed at graphite surface. As revealed by FT‐IR spectra (Figure 5f), the secondary conformation of amyloid peptide was modulated by the GO mediation. Importantly, the typical peaks representing the antiparallel β‐strand secondary conformation almost disappeared; even the β‐strand characteristic was impacted in the presence of GO, and the peak at 1628 cm^−1^ vanished nearly. Furthermore, the content of typical β‐strand secondary conformation of Aβ peptides decreased with the increment of GO amount, which was verified by CD spectra (Figure S6, Supporting Information). Similarly, the structure and the stability of peptide assembly at GO surface were investigated by MD simulation. The assembly formed by 12 Aβ peptides was constructed and the peptides at both ends are restrained to their initial position. Both types of peptide assembly can adsorb to GO surface, while the adsorption of antiparallel orientated assembly is relatively modest (Figure 5g). The average number of peptide heavy atoms in direct contact with GO is 330 for parallel orientated assembly, but 270 for antiparallel orientated assembly (Figure 5h). Meanwhile, the parallel orientated assembly of amyloid peptide at GO surface can maintain its initial packing structure, and the average number of contacted atom pairs in the central region (formed between peptide pairs P_5_‐P_6_, P_6_‐P_7_ and P_7_‐P_8_) is 100, comparable to the peptide assembly on graphene (Figure 5i). On the other hand, the structure fluctuation in antiparallel orientated assembly is considerable. There is one peptide (P_11_) even curls up. The average number of contacted atom pairs in the central region is only 65 (Figure 5j). In short, the parallel orientated assembly can adsorb to GO and maintain its structure, while the adsorption and stability of antiparallel orientated structure appears to be considerably weaker, even though the neighboring peptides can still have hydrophobic interaction with each other. Aβ_33‐42_ peptide tends to form parallel orientated assembly. It is therefore proven from both the experiments and simulations that the monolayer structure is assembled from Aβ_33‐42_ with parallel β‐strand‐like conformation rather than antiparallel β‐strand structures.

The secondary structure of amyloid peptide can be tuned by the 2D carbon interfaces (graphite and GO) to further self‐assemble into molecular monolayer structure rather than amyloid fibrils. In previous research, amyloid fibrils were observed on highly oriented pyrolytic graphite (HOPG) surface from the incubation solution. The assembled lamella structures of amyloid peptides with antiparallel β‐sheet secondary structures were revealed for the proposed mechanism of amyloid fibrillization in the same sample by using STM.^13^ The graphite might be the template for unzipping the amyloid fibril, so that the STM can help to dissolve the assembled structure related to the peptide fibrillization.[[qv: 13,27a]] Herein, amyloid peptides self‐assembled into expanded monolayer through introducing the surface mediator from the initial stage of the aggregation. The secondary structures of peptide assembling were tuned from antiparallel β‐strand for fibrils to parallel β‐strand‐like conformation for molecular layer. In this work, we observe trhe coexistence of a molecular layer (parallel arrangement) and fibril (antiparallel arrangement). The parallel arrangement leads to the molecular layer of amyloid peptide. The molecular layer was the dominant structure accounting for 70.9% surface coverage, which proved the higher degree of the exposure of hydrophobic region. The cell viability of the amyloid early aggregates was investigated further, and it is obviously observed that the early amyloid aggregates presented stronger impact on the cell viability compared to the amyloid fibrils (see Figure S6, Supporting Information). This finding suggests that early aggregates of amyloid peptide Aβ_33‐42_ could be toxic to the cell and the molecular layers we found here are possible aggregates that might be correlated to the cell toxicity.

## 3 Conclusions

The structure of molecular monolayer of Aβ_33‐42_ with parallel β‐strand‐like conformation has accordingly been identified both experimentally and theoretically. It is very different from the Aβ_33‐42_ fibrils with antiparallel β‐strand conformation. The identified molecular monolayer with high degree of hydrophobic region exposure supports the previous finding^27^ and also enriches the polymorphic amyloid assemblies. The proposed self‐assembling mechanism of amyloid peptides in the interface might enlighten the mechanistic insight that the secondary conformation of peptides determines the further assembled nanostructures, and this identified molecular structure in amyloid peptide aggregation might possibly be closely related to the pathogenesis of amyloid disease.

## 4 Experimental Section

*Sample Preparation*: Aβ_33‐42_ (American Peptide Company, USA) was dissolved in hexafluoroisopropanol (HFIP, Sigma) initially for 24 h and then made it dry in vacuum. The sample treated above was dissolved in MilliQ water to the concentration of 100 × 10^−6^ m incubated for 1 h. This solution (25 μL) was then dropped onto freshly cleaved HOPG surface and characterized by quantitative nanomechanical mapping. Measurements were carried out at ambient conditions.

*Quantitative Nanomechanical Mapping*: The quantitative nanomechanical mapping is AFM‐based force spectroscopy routinely used for quantitative mechanical property measurements and submolecular resolution imaging of biological samples. The experiments were performed with a commercial AFM instrument (Multimode V, Bruker, USA) in harmonic torsion mode at ambient conditions. T‐shaped torsional harmonic cantilever was used for nanoscale mechanical property mapping. The resonant frequency of cantilever was 62.3 kHz; the quality factor was ≈50. The deflection sensitivity of the tip was calibrated to be 36.70 nm V^−1^ and the spring constant was determined to be 0.56 N m^−1^. Before mapping the cantilever was calibrated by ramp and thermal tuning. The time‐varying tip‐sample force waveforms and the effective elastic modulus were calculated using our previously described mathematical procedure.[[qv: 14a,21]] The interactions between the tip and sample were determined by the long‐range electrostatic and van der Waals (VDW) forces, and short‐range mechanical restoration forces. The following formula derived for a spherical tip indenting a semi‐infinite planar sample was used to estimate a local reduced elastic modulus:

$$F_{\mathrm{interaction}}=(4/3)E^{*}\sqrt{R}{\left(d-d_{0}\right)}^{3/2}+F_{\mathrm{adh}}$$

where $F_{\mathrm{interaction}}$ is the tip‐sample force, $E^{*}$ the reduced elastic modulus of the tip and the sample, *R* the tip radius, $d_{0}$ the surface rest position, $d-d_{0}$ the depth of indentation, and $F_{\mathrm{adh}}$ the constant adhesion force during the contact. All the topography, elastic modulus, and force maps were analyzed by using the commercial software Scanning Probe Image Processor (Image Metrology, Denmark).

*Turbidity Measurements*: Turbidity was measured on a fluorescence spectrophotometer at room temperature (PerkinElmer LS55) by using 1 cm path‐length quartz cell. Both excitation and emission wavelengths were set to 400 nm with spectrum band width of 1 nm. The signal was quantified by averaging the emission intensity at 400 nm (slit width = 2.5 nm) over 15 s in an attenuate mode.

*Fourier Transform Infrared Spectroscopy*: FT‐IR spectra were recorded on Spectrum Two with UATR (Single Reflection Diamond) accessory (PerkinElmer, Waltham, MA, USA). Spectra were obtained from 32 scans at 4 cm^−1^ resolution.

*Synchrotron Radiation Circular Dichroism*: Beamline CD1 at the ASTRID storage ring (ISA, Aarhus University, Denmark) was used to collect the SRCD spectra. The beam from CD1 (Miles2007, Miles2008) was polarized with a MgF_2_ Rochon polarizer (B‐Halle GmbH, Berlin), and a photoelastic modulator (Hinds, USA) produced alternating left and right‐handed circular polarized light. The Aβ_33‐42_ samples were measured at concentrations of 200, 100, and 50 × 10^−6^ m dissolved in MilliQ water incubated for 1.5 h after dissolving it in HFIP (Sigma) initially for 24 h. The light passed through the sample and was detected by a photomultiplier tube (Type 9406B, ETL, UK). Samples were measured in 1 mm path‐length Suprasil cells (Hellma GmbH) and spectra of the water were recorded as baseline subtraction. All samples and baseline spectra were collected in triplicate with 1 nm step size and 2 s dwell time. The spectra were averaged, baseline subtracted, and mildly smoothed with a Savitzky–Golay filter using the CD tool software.[[qv: 20a]]

*Theoretical Calculations*: Terminal interaction of amyloid peptide determines the basic building block in amyloid peptide assembling structures. The possible conformations based on two termini peptides as a unit were calculated. Furthermore, the network formation based on the units was also calculated and optimized. All the calculations were performed using the Grid‐Based Projector‐Augmented Wave (GPAW) code. The structures were optimized using the Perdew–Burke–Ernzerh^28^ functional. In all calculations, the standard GPAW setups for hydrogen, carbon, oxygen, and nitrogen atoms were used, and the energies were converged to a threshold of 0.001 eV per atom.

*Molecular Dynamic Simulation*: The peptide assemblies adsorbed on both graphene and graphene oxide were constructed. The peptide assembly was composed of 12 peptides (peptide A to L) with the interpeptide separation of 7.55 Å. The stabilities of the assemblies with parallel and antiparallel packing structure were studied. And the peptides at both ends (peptide A and L) were restrained to their initial positions during the simulations. The simulations were performed by using NAMD 2.8 program. CHARMM27 all‐atom force field was used in the simulation. The water model was TIP3P. VDW interactions were treated by the switch function with twin‐range cutoff distances of 10 and 12 Å, and the electrostatic interactions were calculated by particle mesh Ewald method with a cutoff distance of 12 Å. All the simulations were carried out at constant temperature (*T* = 300 K), and the Langevin thermostat method was used with the damping coefficient of 1 ps^−1^. All the snapshots were rendered with VMD software.

## Supporting information

As a service to our authors and readers, this journal provides supporting information supplied by the authors. Such materials are peer reviewed and may be re‐organized for online delivery, but are not copy‐edited or typeset. Technical support issues arising from supporting information (other than missing files) should be addressed to the authors.

Supplementary
