# Supplementary material for: Identification of a Novel Parallel β‐Strand Conformation within Molecular Monolayer of Amyloid Peptide
Source: Adv Sci (Weinh). 2016 Apr 8;3(6):1500369. doi: 10.1002/advs.201500369 (PMC5071675; doi:10.1002/advs.201500369)
Supplement: Supplementary file 1 — Supplementary [file ADVS-3-0h-s001.pdf]

Copyright WILEY-VCH Verlag GmbH & Co. KGaA, 69469 Weinheim, Germany, 2016.

# Supporting Information

for *Adv. Sci.,* DOI: 10.1002/advs.201500369

# Identification of a Novel Parallel *β*-Strand Conformation within Molecular Monolayer of Amyloid Peptide

*Lei Liu, Qiang Li, Shuai Zhang, Xiaofeng Wang, Søren Vrønning Hoffmann, Jingyuan Li, Zheng Liu, Flemming Besenbacher,* and Mingdong Dong**

Copyright WILEY-VCH Verlag GmbH & Co. KGaA, 69469 Weinheim, Germany, 2013.

# Supporting Information

## Identification of a novel parallel β-stand conformation within molecular monolayer of amyloid peptide

*Lei Liu, Qiang Li, Shuai Zhang, Xiaofeng Wang, Søren Vrønning Hoffmann, Jingyuan Li, Zheng Liu, Flemming Besenbacher*, Mingdong Dong**

Figures. S1 to S6
Tables S1 and S2

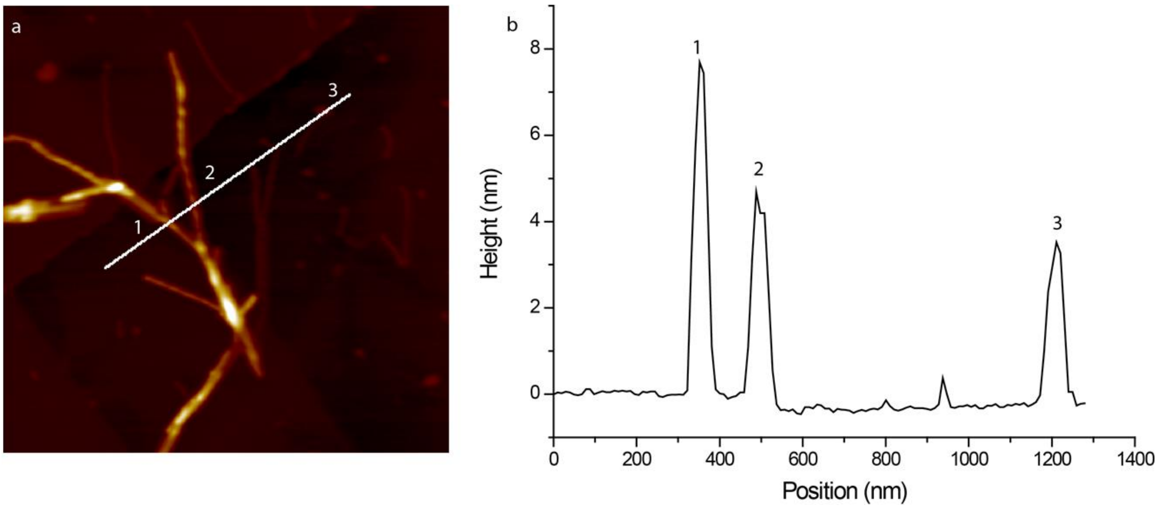


**Figure. S1. The topography image and height line profile of amyloid peptide A$\beta_{33\text{-}42}$.** (a) The topography image of amyloid peptide fibrils and oligomers. The white line indicates the amyloid fibril and oligomer measured. (b) The height line profile was plotted. Peaks 1 and 2 show the heights of amyloid fibrils at 7.8 nm and 4.9 nm, respectively. Peak 3 shows the height of amyloid oligomer at about 4 nm.

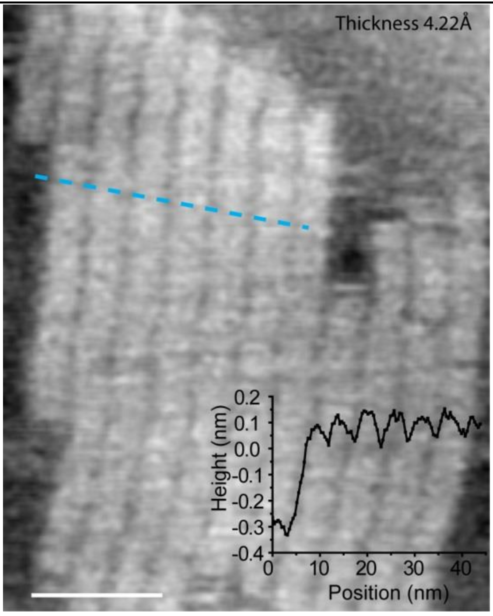


**Figure. S2. The topography image of nanostriped structures consisting of amyloid peptide A$\beta_{33\text{-}42}$.** The continuous nanostripes are observed by quantitative nanomechanical mapping technique. The inset line profile presented in the lower panel corresponds to the blue dashed line across the nanostripes. The height of the nanostripes is approximately 0.45 nm which is consistent with the peptide width. The scale bar of the topography image is 20 nm.

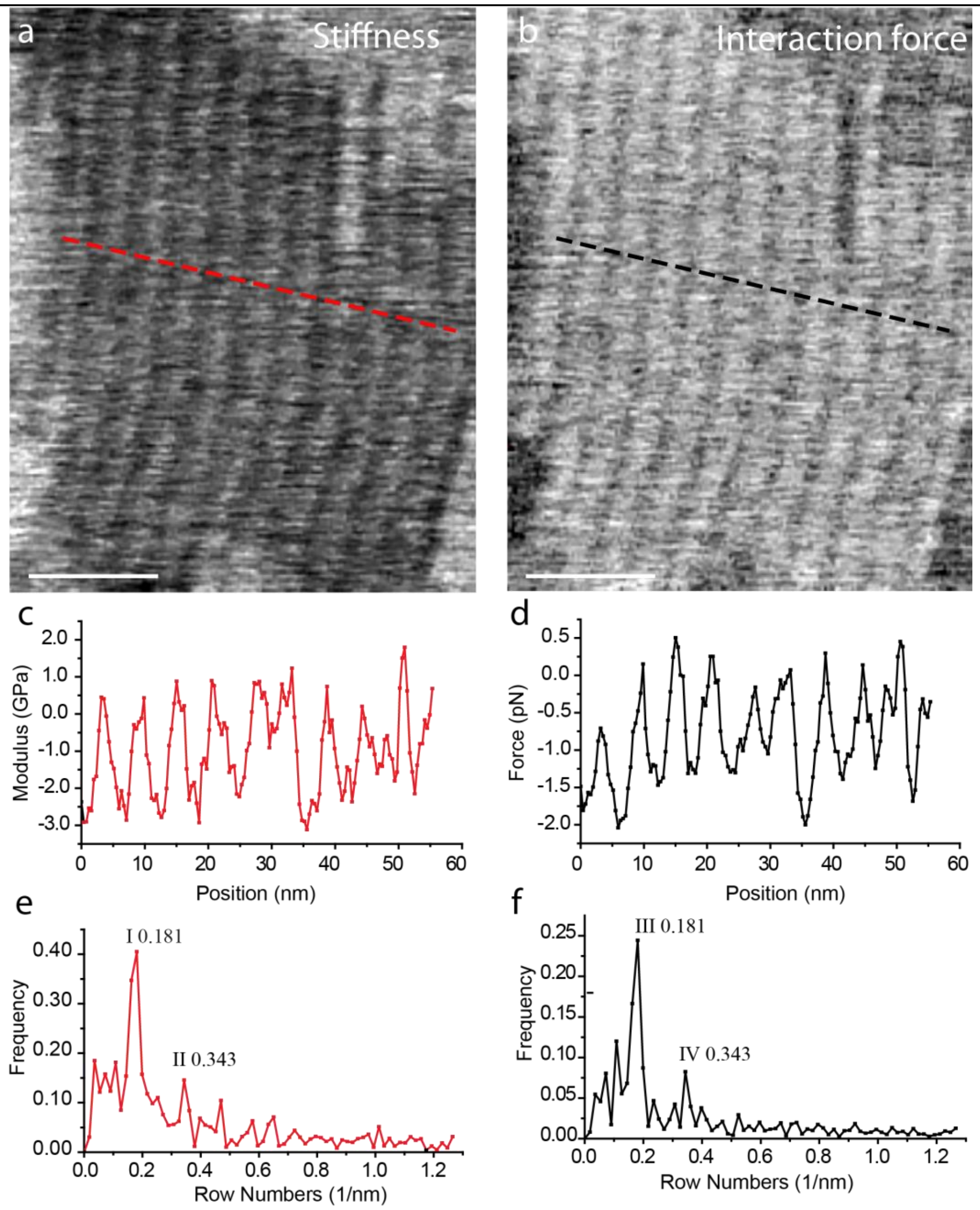


**Figure. S3. The stiffness map and interaction force map of nanostripe structures consisting of amyloid peptide A$\beta_{33\text{-}42}$.** (a) The stiffness map of amyoid peptide nanostripes. (b) The interaction force map of amyloid peptide nanostripes. (c) and (d), The line profiles of stiffness and interaction force of amyloid peptide nanostripes according to the red and black dashed lines in (a) and (b). (e) and (f) The Fourier transformation analysis of stiffness and interaction force maps of nanostriped structures under an applied force $F_2$. The two peaks in (e) and (f) indicate that there are two periodicities in the nanostriped structures. The periodicities of the nanostripes are calculated to be 5.5 nm and 2.9 nm both for stiffness and interaction force, respectively. The scale bars of (a) and (b) are 20 nm.

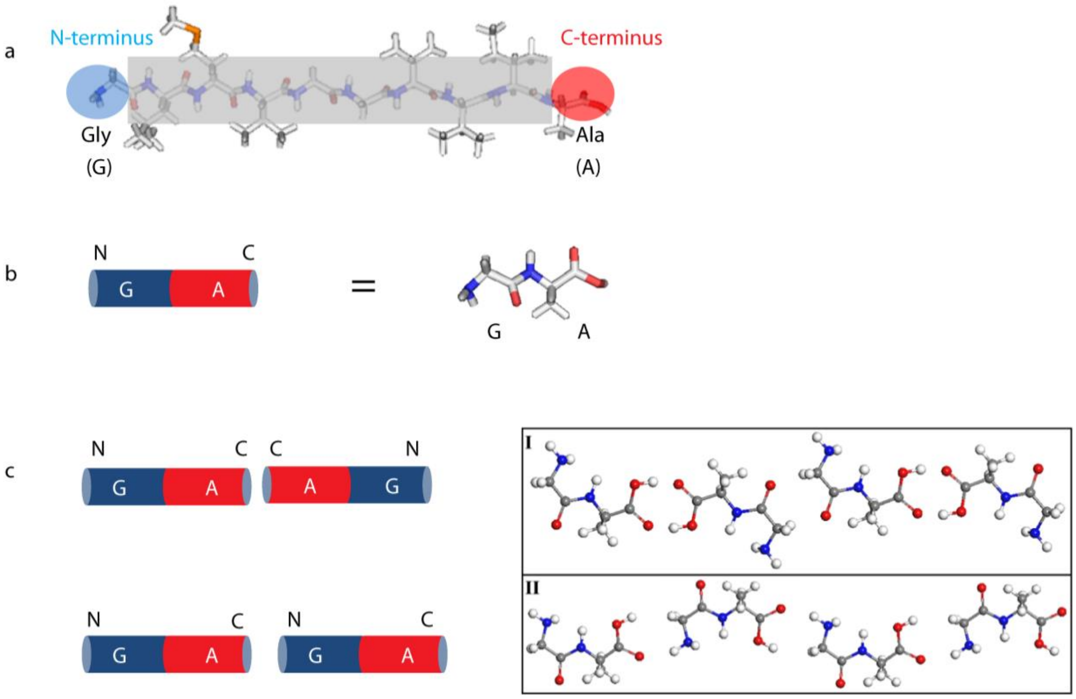


**Figure. S4. The interaction models of peptide termini.** (a) The structure model of amyloid peptide Aβ33-42. The grey part is eight residues in the backbone of peptide, and the glycine and alanine locate at the terminus, which are marked with blue and red ball, respectively. (b) The schematic model of dipeptide glycine and alanine representing the whole amyloid peptide when we calculating the interaction of two peptides considered that terminus interaction of peptide give great contribution to the assembling structure formation. (c) The two interaction models of peptide termini. One is the interaction model of carboxylic termini of peptide, and the peptide prefers to form dimer structures *via* the interactions of C termini in marked "I'. The other one is the interaction model of carboxylic terminus and nitrogen terminus of peptide in marked "II". The model II is less stable than model I. The grey, red and blue atoms represent the carbon, oxygen and nitrogen atoms, respectively. The hydrogen bond distance of O-H···O in C-C system is about 1.60 Å, which is also shorter than that of O···H-N (2.24 Å) in C-N system. The optimum network is C-C configuration (Fig. S4.c), which is in good agreement with AFM image contrast. Thus, a C-C dimer in a single stripe with 5.50 ± 0.10 nm periodicity is the most preferential complex formed by peptide by surface mediation.

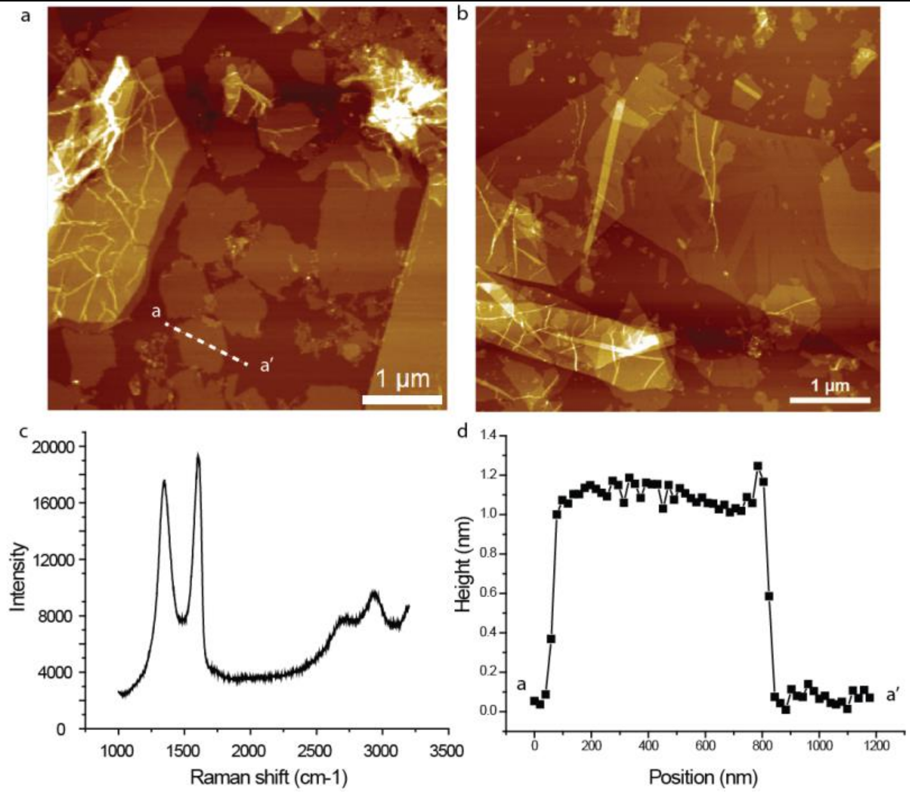


**Figure. S5. AFM and Raman characterization of GO.** (a) The AFM image of GO on mica surface. (b) The complex of amyloid peptide and GO was observed by AFM. The monolayer carpet of peptide was clearly observed on top of GO. (c) The Raman spectra of GO. (d) The height profile of monolayer GO.

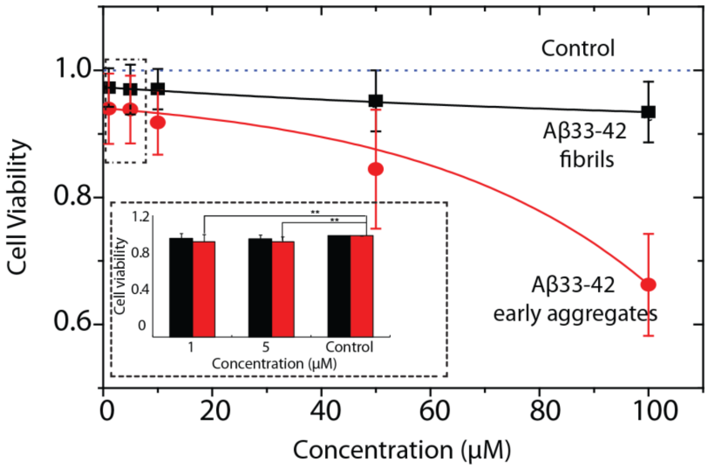


**Figure S6**. The cell viability of amyloid early aggregates and amyloid fibrils, respectively, plotted as a function of the target concentration. The zoom-in plot presents the cell viability of amyloid peptides at low concentrations with fibrils and early aggregates. Incubating conditions, 10 mM PBS buffer solution and pH = 7.4. Results are mean ± SD (n = 3). Statistical differences compared with the controls are given as *, $P < 0.05$ and **, $P < 0.01$.

**Table S1.** The periodicity of amyloid peptide nanostripe structure based on Fourier transformation analysis under different applied forces in height, modulus and interaction force maps. The value of row number represents the peak in original Fourier transformation data. The periodicity can be calculated corresponding to the row number. Two sets of data are in this table under force $F_1$ and $F_2$, respectively.

| Data type | | Height | | Modulus | | Interaction Force | |
|---|---|---|---|---|---|---|---|
| | | Row number (1/nm) | Periodicity (nm) | Row number (1/nm) | Periodicity (nm) | Row number (1/nm) | Periodicity (nm) |
| Applied Force | $F_1$ | 0.186, 0.372 | 5.38, 2.69 | 0.186, 0.372 | 5.38, 2.69 | 0.186 | 5.38 |
| | $F_2$ | 0.178, 0.356 | 5.62, 2.81 | 0.181, 0.343 | 5.53, 2.92 | 0.181, 0.343 | 5.53, 2.92 |

**Table S2.** The binding energy and distance of the interactions between the peptide termini. The binding energy of two, four, six and eight amyloid peptides *via* C-terminal interactions and *via* C- and N-terminus interactions are displayed in the table, respectively. Distance of O-H----O and O----H-N in two, four, six and eight amyloid peptides are also displayed in the table, respectively.

| Number of protein | Energy (eV) | | Distance (Å) | |
|---|---|---|---|---|
| | C-C | C-N | O-H----O | O----H-N |
| **Two** | -0.39 | -0.06 | 1.60 | 2.24 |
| **Four** | -0.44 | -0.08 | 1.61 | 2.22 |
| **Six** | -0.44 | -0.09 | 1.60 | 2.25 |
| **Eight** | -0.45 | -0.09 | 1.60 | 2.23 |
